# Fatigue in Chinese Patients With Amyotrophic Lateral Sclerosis: Associated Factors and Impact on Quality of Life

**DOI:** 10.3389/fneur.2022.806577

**Published:** 2022-02-18

**Authors:** Ran An, Cheng Li, Xin Li, Yuan Wu, Xianghua He, Shaolong Ai, Yanming Xu, Chengqi He

**Affiliations:** ^1^Key Laboratory of Rehabilitation Medicine in Sichuan, Department of Rehabilitation Medicine, West China Hospital, Sichuan University, Chengdu, China; ^2^Department of Neurology, West China Hospital, Sichuan University, Chengdu, China

**Keywords:** fatigue, amyotrophic lateral sclerosis, ALS, QOL, Quality of Life

## Abstract

**Objectives:**

Fatigue was considered as a common symptom in amyotrophic lateral sclerosis (ALS). Previous studies about the impact of fatigue on Quality of Life (QoL) in patients with ALS were limited and inconsistent. Besides, a systematic investigation of fatigue in Chinese patients with ALS was lacking. Therefore, this study aimed to comprehensively evaluate the frequency and associated factors of fatigue and impact on QoL in Chinese patients with ALS.

**Participants and Methods:**

Probable and definitive patients with ALS and age- and gender-matched healthy controls (HCs) were consecutively recruited. The frequency of fatigue between both the groups was determined by the Fatigue Severity Scale (FSS). Disease severity, sleep quality, sleepiness, anxiety, depression, and QoL were evaluated in patients with ALS by the ALS Functional Rating Scale-revised (ALSFRS-R) and the ALS Severity Scale (ALSSS), the Pittsburgh Sleep Quality Index (PSQI), the Epworth Sleepiness Scale (ESS), the Hamilton Anxiety Rating Scale (HARS), the Hamilton Depression Rating Scale (HDRS), and the McGill Quality of Life Questionnaire (MQOL). Then, clinical characteristics of patients with ALS with fatigue were compared with those without fatigue. Lastly, associated factors of fatigue and impact on QoL in Chinese patients with ALS were assessed.

**Results:**

A total of 175 patients with ALS and 175 HCs were included. Fatigue was significantly more frequent in patients with ALS than in controls (32.6 vs. 17.7%, *p* = 0.001). Patients with ALS with fatigue scored lower on the ALSFRS-R, the ALSSS [lower extremity (LE) + upper extremity (UE)], the total ALSSS, higher in the HARS, HDRS, PSQI, ESS scores, and a poorer QoL. Daytime dysfunction and the ALSSS (LE + UE) score were associated with a higher risk of fatigue. The ALSSS (LE + UE), the FSS, age, the HARS, and the HDRS score were significantly associated with various aspects of QoL.

**Conclusion:**

This study has described fatigue in Chinese patients with ALS and finding daytime dysfunction and the lower ALSSS (LE + UE) were associated with a higher risk of fatigue. Also, we identified an inverse relationship of fatigue intensity with the psychological domain of QoL.

## Introduction

Amyotrophic lateral sclerosis (ALS) is a neurodegenerative disorder of unknown etiology, characterized by the progressive loss of upper and lower motor neurons, causing weakness and atrophy of upper and lower limbs, dysphagia, and dysarthria, eventually resulting in death due to respiratory failure typically within 2–4 years from disease onset (1). No curative treatment is available for patients with ALS, so the paramount consideration in clinical practice is symptom relief to improve Quality of Life (QoL) (2).

Quality of Life in patients with ALS was affected not only by the physical manifestations but also by the psychosocial effects of living with a terminal, disabling illness (3).

Fatigue was a common and understudied symptom in patients with ALS, affecting 44–86% of patients with ALS (4–7). Fatigue has been defined as an overwhelming sense of tiredness, lack of energy, and feeling of exhaustion (8). It has an important impact on QoL in patients with ALS (4, 9, 10). Fatigue in ALS has been reported to be associated with age, site of onset, functionality severity, disease duration, and disease progression (5, 6, 11, 12), and forced vital capacity, excessive daytime sleepiness (EDS), depression, and poor quality of sleep (11). The causes and mechanisms of fatigue in ALS can be multifactorial, including both central and peripheral fatigue (8). Fatigue in ALS, as in other neurological diseases, can reduce physical endurance and functionality, affecting daily life activities, QoL, and familial, professional, and social interactions (13). Researches about the impact of fatigue on QoL in patients with ALS were limited and inconsistent (4, 5, 9, 10). To our knowledge, there has been no study to systematically examine fatigue in patients with ALS from China.

Therefore, the objective of this study was to comprehensively investigate the frequency and associated factors of fatigue and impact on QoL in a large group of Chinese patients with ALS, with special attention to the relationship between fatigue and QOL.

## Methods

### Participants

This study was conducted in a tertiary referral center in Southwest China (West China Hospital of Sichuan University) from the year 2020 to 2021. Patients were eligible and consecutively recruited if they fulfilled the criteria of probable and the definitive ALS according to the revised El Escorial criteria (14). Patients were excluded if they were unable to give informed consent or unable to complete self-report questionnaires even with help. Also, the Mini-Mental Status Examination (MMSE) was used to screen the cognition. Patients were not eligible if they had cognitive impairment or significant concomitant conditions precluding them from being able to select responses on questionnaires. We collected detailed clinical data of eligible patients with ALS during their first visit to our hospital.

Normal healthy controls (HCs) (relatives of patients in general), without neurological or other associated diseases, were recruited to match patients with ALS according to gender and age.

### Procedure

First, the demographic and clinical data of patients with ALS and HCs included in this study were collected, as needed. The evaluation of fatigue between patients with ALS and normal HCs was performed by the Fatigue Severity Scale (FSS). Disease severity, sleep quality, sleepiness, anxiety, depression, and QoL were assessed in patients with ALS by validated Chinese translation questionnaires of the ALS Functional Rating Scale-revised (ALSFRS-R), the ALS Severity Scale (ALSSS), the Pittsburgh Sleep Quality Index (PSQI), the Epworth Sleepiness Scale (ESS), the Hamilton Anxiety Rating Scale (HARS), the Hamilton Depression Rating Scale (HDRS), and the McGill Quality of Life Questionnaire (MQOL). Then, clinical data of patients with ALS with fatigue were compared with those without fatigue. Lastly, associated risk factors of fatigue and impact on QoL in patients with ALS were analyzed.

### Assessments

#### Physical Functional Status and Disease Severity Assessments

The physical functional status of patients with ALS was evaluated with the ALSFRS-R, a 12-item scale assessing bulbar, limb, and respiratory function. Each item is rated from 0 (worse) to 4 (best), corresponding to a total score ranging from 0 to 48, with higher scores indicating greater physical status and function (15). ALSFRS-R limbs (ALSFRS-Rl) was calculated by summing the subscales of ALSFRS-R upper limbs (ALSFRS-Rul) and ALSFRS-R lower limbs (ALSFRS-Rll). ALSFRS-Rul and ALSFRS-Rll were obtained by scoring the fourth and fifth questions for the former and the eight and ninth for the latter.

Amyotrophic Lateral Sclerosis Severity Scale (ALSSS) has been developed to provide an ordinal staging system and a means of rapid functional assessment for patients with ALS. The scale allows an examiner to evaluate the symptoms of patients with ALS numerically in four categories that describe speech (SP), swallowing (SW), lower extremity (LE), and upper extremity (UE) abilities. Each section has ten possible rating scores based on the progressive decline of function in one specific area. In addition to each rating from 1 to 10, the bulbar function can be described by adding the scores for SP and SW. Similarly, the spinal function can be described by adding the scores for LE and UE. A total score can be assigned by adding the four sub-scores (SP + SW + LE + UE) (16). We also collected the following clinical data for each patient with ALS included in the study: gender, age, age at onset, site of onset, disease duration, dyspnea, or not.

#### Sleep Quality, Excessive Daytime Sleepiness Assessments

Sleep quality was assessed using the PSQI. The PSQI score >5 was taken to indicate poor sleep (17). Daytime somnolence was assessed using the ESS. The ESS assesses how sleepy a subject is while doing different tasks. Total possible scores range from 0 (not sleepy at all) to 24 (very sleepy). Excessive daytime sleepiness was defined by the ESS score of ≥ 10 (18).

#### Fatigue Assessments

Fatigue was evaluated by the FSS. The FSS is a simple and reliable questionnaire used to assess and quantify fatigue for clinical and research purposes. The FSS is a self-reported fatigue questionnaire with nine items measuring subjective fatigue in the preceding 2 weeks. Participants were asked to rate the degree of fatigue on a scale of 1 (strongly disagree) to 7 (strongly agree). The average score of the nine items was recorded as the FSS score; the high FSS score indicates a high level of fatigue intensity. Clinically significant fatigue is defined as the FSS score ≥ 4 (19).

#### Depression and Anxiety Assessments

Depression and anxiety were assessed using the 24-item HDRS and the 14-item HARS, respectively. The HDRS consisted of 24 items, the severity of each item was rated from 0 (not present) to 4 (very severe) or 2 (present) and the scores ranged from 0 to 76 (20). The HARS included 14 items, symptom severity of each item was rated from 0 (not present) to 4 (very severe), and the scores ranged from 0 to 56 (21). The HDRS scores >8 indicated possible depression, >20 depression; The HARS scores >7 indicated possible anxiety, >14 anxiety.

#### Quality of Life Assessment

The MQOL was used to measure multiple dimensions and overall QOL in patients with ALS. The MQOL is a 17-item [16 items and a single-item scale (MQOL-SIS)] multidimensional tool. There are five MQOL subscales, which are specifically designed to measure physical wellbeing, physical symptoms, psychological symptoms, existential wellbeing, and social support. The MQOL incorporates the existential domain, balances physical and non-physical aspects of QoL, and includes both positive and negative influences on QoL. Each question uses a 0–10 scale. Each subscale score is the mean of the items forming that subscale. The MQOL total (summary) score is the mean of five subscale scores (22, 23).

This study was approved by the Institutional Ethics Committee of West China Hospital, Sichuan University (approval 2020-942). All the participants were informed of the purpose of this study before enrollment and gave a written informed consent for participation.

#### Statistical Analysis

Continuous parameters that were normally distributed were described as the means ± SD. Those with a non-normal distribution were presented as median and interquartile range (IQR). Categorical variables were summarized using patient counts and percentages. The Student's *t*-test (with Mann-Whitney *U* test when necessary) was used to compare continuous variables and the chi-squared test was used to analyze categorical variables.

As there were four partially overlapping questionnaires dealing with similar sleep symptoms, namely, the HAMA, the HDRS-24, the PSQI, and the ESS, the Bonferroni correction was applied, and *p*-value was set at 0.0045 (0.05/11) for testing the HARS, the HDRS, the PSQI (including seven subscores), and the ESS in the univariate analysis.

The binary logistic regression analysis (forward stepwise LR method) was used to evaluate the association between the variables and fatigue. The risk factors for fatigue in patients with ALS were investigated by univariate statistical analysis and the results were controlled using a binary logistic regression model. Variables with statistical significance in the univariate analysis or considered clinically relevant were included in the multivariate analysis. The Box-Tidwell test was used for the assumption of linearity in the logit for the continuous variable. Multicollinearity was assessed using an examination of the variance inflation factor in a multiple regression model with the same variables. The results were expressed as odds ratios (OR) with their corresponding 95% CIs. The sample size in this study met the demands of event per variable (EPV) during the logistic regression analysis.

Variables correlated with fatigue intensity and QoL were considered in a multiple linear stepwise regression model. Data were tested for linearity, independence of residuals, homoscedasticity, and normality, and lack of multicollinearity and large leveraged values. A two-tailed *p*-value < 0.05 was considered as statistical significant. All the statistical analyses were performed using the SPSS version 25.0 (SPSS Incorporation, Armonk, New York, USA).

## Results

### Demographic Characteristics in Patients With ALS and HCs

A total of 175 patients with ALS (105 males and 70 females) and 175 HCs (95 males and 80 females) were included in this study, with a median age of 54 years old (IQR: 47–63) and 53 years old (IQR: 44–61), respectively, without significant difference. There were significant statistical differences in the presence of fatigue (the FSS score ≥ 4) between patients with ALS and the HC groups (*p* = 0.001). A comparison of demographical and FSS scores between patients with ALS and HCs is shown in Table 1.

**Table 1:** Demographic and clinical characteristics of patients with ALS and healthy controls.

	**ALS patients**	**Healthy controls**	***p*-Value**
	**(*n* = 175)**	**(*n* = 175)**	
Gender (F: M)	70:105	80:95	0.280
Age at interview, year	54 (47–63)	53 (44–61)	0.243
FSS	2.6 (1.2–4.6)	3.0 ± 1.0	0.128
FSS ≥ 4	57 (32.6%)	31 (17.7%)	**0.001**

*F, female; M, male; FSS, Fatigue Severity Scale. The Mann-Whitney U test was used to compare continuous variables and the chi-squared test was used to analyze categorical variables between both the groups. The meaning of the bold values represented statistically significant values in the statistical analysis*.

### Clinical Characteristics in Patients With ALS With Fatigue and Without Fatigue

A total of 57 patients with ALS (32.6%), namely, 34 males (59.6%) and 23 females (40.4%), reported clinically significant fatigue.

There were no statistical differences in gender, age at interview, age at onset, site of onset, disease duration, the ALSSS (SP + SW) score between patients with ALS with fatigue, and patients without fatigue (*p* > 0.05). Patients with ALS with fatigue scored lower on the ALSFRS-R, the ALSFRS-Rl, the ALSSS (LE + UE), and the ALSSS (LE + UE + SP + SW), compared with patients without fatigue (*p* < 0.001, *p* < 0.001, *p* < 0.001, *p* = 0.001, respectively).

Also, patients with ALS with fatigue had the higher HDRS and the ESS scores (*p* < 0.001, < 0.001, respectively), and on subitems of sleep quality, sleep disturbances, and daytime dysfunction (*p* = 0.004, 0.002, and < 0.001, respectively).

A comparison of demographical and clinical data between patients with ALS with fatigue and without fatigue is shown in Tables 2, 3.

**Table 2:** Demographic and clinical characteristics of patients with ALS with fatigue or without fatigue.

	**All ALS patients** ** (*n* = 175)**	**ALS patients with fatigue** ** (*n* =57)**	**ALS patients without fatigue** ** (*n* =118)**	***p*-Value**
Gender (F: M)	70:105	23:34	47:71	0.947
Age at interview, year (SD)	54 (47–63)	55.3 ± 10.7	52 (45.8–63.3)	0.153
Age at onset, year (SD)	52 (46–62)	54.3 ± 10.6	52 (44.8–62)	0.155
Spinal onset, *n* (%)	132 (75.4%)	45 (78.9%)	87 (73.7%)	0.452
Disease duration (m)	11 (7–16)	12 (8.5–18)	10 (6–14)	0.185
Dyspnea	63 (36%)	25 (43.9%)	38 (32.2%)	0.132
ALSFRS-R score	40 (35–43)	36 (32–41)	40.5 (37–44)	**<0.001**
ALSFRS-Rl	12 (9–14)	10 (7.5–13)	12 (10–14)	**<0.001**
ALSFRS-Rb	11 (9–12)	11 (9–12)	11 (9–12)	0.399
ALSSS (LE + UE)	16 (13–18)	13.5 ± 3.6	16 (14–18)	**<0.001**
ALSSS (SP + SW)	19 (14–20)	19 (13.5–20)	19 (14–20)	0.578
Total ALSSS (LE + UE + SP + SW)	33 (29–36)	30 (28–34)	34 (30–36.3)	**0.001**

*F, female; M, male; disease duration (month) was defined from the onset of symptoms; m, month; ALSFRS-R, Amyotrophic Lateral Sclerosis Functional Rating Scale-Revised; ALSFRS-Rl, Amyotrophic Lateral Sclerosis Functional Rating Scale-Revised Limbs; ALSFRS-Rb, Amyotrophic Lateral Sclerosis Functional Rating Scale-Revised Bulbar; ALSSS, Amyotrophic Lateral Sclerosis Severity Scale; SP, speech; SW, swallowing; LE, lower extremity; UE, upper extremity. The Mann-Whitney U test was used to compare continuous variables and the chi-squared test was used to analyze categorical variables between both the groups. The meaning of the bold values represented statistically significant values in the statistical analysis*.

**Table 3:** Differences in non-motor syndromes and quality of life between patients with ALS with fatigue and without fatigue.

	**ALS patients with fatigue ** ** (*n* = 57)**	**ALS patients without fatigue** ** (*n* = 118)**	***p*-Value**
HARS	9 (3.5–16)	6 (3–10.3)	0.006
HARS > 7, *n* (%)	34 (59.6%)	40 (33.9%)	**0.001**
HARS > 14, *n* (%)	16 (28.1%)	17 (14.4%)	0.03
HDRS	11 (6.5–17)	7 (3–11)	**<0.001**
HDRS > 8, *n* (%)	38 (66.7%)	46 (39%)	**0.001**
HDRS > 20, *n* (%)	10 (17.5%)	1 (0.8%)	**<0.001**
MMSE	27 (26.5–28.5)	28 (26–29.5)	0.154
Sleep quality	1 (1,2)	1 (1)	**0.004**
Sleep latency	2 (0–3)	1 (0–2)	0.172
Sleep duration	1 (1–3)	1 (0–2)	0.193
Sleep efficiency	1 (0–3)	1.5 (0–3)	0.960
Sleep disturbances	1 (1,2)	1 (1)	**0.002**
Use of hypnotics	0 (0–0)	0 (0–0)	0.027
Daytime dysfunction	2 (1–3)	0 (0–1)	**<0.001**
PSQI	8 (5–15)	7 (4–10)	0.011
PSQI > 5, *n* (%)	40 (70.2%)	71 (60.2%)	0.198
ESS	6 (3–10)	3 (1–6)	**<0.001**
ESS ≥ 10	17 (29.8%)	13 (11.0%)	**0.002**
FSS	5.3 (4.6–6.3)	1.7 (1–2.6)	**<0.001**
MQOL-SIS	6 ± 2.4	7 (6–8)	**0.007**
MQOL-PhS	4.9 ± 2.9	6.7 (3.9–8.3)	**0.020**
MQOL-PhWB	5 (3–6.5)	5 (3–8)	0.105
MQOL-PsyS	4.8 (3.5–7.3)	7.3 (5.5–8.6)	**<0.001**
MQOL-EWB	5.8 ± 1.9	6.3 (4.8–8.2)	0.157
MQOL-SS	7.5 (5–8)	8 (6.5–9.5)	**0.004**
MQOL-Total	18 ± 5.3	20.5 ± 4.6	**0.004**

*HARS, Hamilton Anxiety Rating Scale; HDRS, Hamilton Depression Rating Scale; PSQI, Pittsburgh Sleep Quality Index; ESS, Epworth Sleepiness Scale; FSS, Fatigue Severity Scale; MQOL, McGill Quality of Life Questionnaire; MQOL-SIS, MQOL single-item scale; MQOL-PhS, MQOL-physical symptoms; MQOL-PhWB, MQOL-Physical wellbeing; MQOL-PsyS, MQOL-Psychological symptoms; MQOL-EWB, MQOL-Existential wellbeing; MQOL-SS, MQOL-S Social support. The Student's t-test (with Mann-Whitney U test when necessary) was used to compare continuous variables and the chi-squared test was used to analyze categorical variables. The meaning of the bold values represented statistically significant values in the statistical analysis*.

### Factors Associated With the Presence of Fatigue

The ALSFRS-R, the ALSFRS-Rl, the ALSSS, the ALSSS (LE + UE), the HADR, the HARS, sleep quality, sleep disturbances, daytime dysfunction, and the ESS scores were included in the binary logistic regression analysis (forward stepwise LR method).

The ALSSS (LE + UE), the ESS, and daytime dysfunction were found to be associated with a higher risk of fatigue (*p* = 0.002, 0.045, and 0.001, respectively) (Table 4).

**Table 4:** Factors associated with the presence of fatigue in the binary logistic regression analysis.

**Covariates**	**B**	**OR**	**95% CI**	***p*-Value**
ALSFRS-R				0.938
ALSFRS-Rl				0.460
ALSSS				0.969
ALSSS (LE + UE)	−0.174	0.840	(0.753,0.938)	**0.002**
HARS				0.796
HDRS				0.130
Sleep quality				0.400
Sleep disturbances				0.201
Daytime dysfunction	0.610	1.840	(1.294,2.617)	**0.001**
ESS	0.082	1.085	(1.002,1.175)	**0.045**

*ALSFRS-R, Amyotrophic Lateral Sclerosis Functional Rating Scale-revised; ALSFRS-Rl, Amyotrophic Lateral Sclerosis Functional Rating Scale-revised limbs; ALSSS, Amyotrophic Lateral Sclerosis Severity Scale; LE, lower extremity; UE, upper extremity; HARS, Hamilton Anxiety Rating Scale; HDRS, Hamilton Depression Rating Scale; ESS, Epworth Sleepiness Scale; OR, odds ratio. The binary logistic stepwise regression analysis (forward stepwise LR method) was used. The meaning of the bold values represented statistically significant values in the statistical analysis*.

### Factors Associated With Fatigue Intensity

Besides the 10 possible factors above, age, gender, age at onset, site of onset, and disease duration was also added in the multiple linear regression model to find clinical variables related to fatigue severity.

The relationship between the FSS score and the ALSSS (LE + UE), daytime dysfunction, and the HDRS scores (*p* = 0.003, < 0.001, and 0.031, respectively) was found (Table 5).

**Table 5:** Factors associated with the intensity of fatigue in the multiple linear regression analysis.

**Covariates**	**B**	**Beta**	**95% CI**	***p*-Value**
ALSFRS-R				0.763
ALSFRS-Rl				0.137
ALSSS				0.651
ALSSS (LE + UE)	−0.122	−0.219	(−0.201, −0.044)	**0.003**
HARS				0.477
HDRS	0.045	0.166	(0.004,0.086)	**0.031**
Sleep quality				0.447
Sleep disturbances				0.787
Daytime dysfunction	0.594	0.339	(0.348,0.839)	**<0.001**
ESS				0.191
Age				0.965
Gender				0.593
Age at onset				0.886
Site of onset				0.679
Disease duration				0.904

*ALSFRS-R, Amyotrophic Lateral Sclerosis Functional Rating Scale-revised; ALSFRS-Rl, Amyotrophic Lateral Sclerosis Functional Rating Scale-revised limbs; ALSSS, Amyotrophic Lateral Sclerosis Severity Scale; LE, lower extremity; UE, upper extremity; HARS, Hamilton Anxiety Rating Scale; HDRS, Hamilton Depression Rating Scale. A multiple linear stepwise regression model was used. The meaning of the bold values represented statistically significant values in the statistical analysis*.

### Impact of Clinical Variables on QoL

The MQOL-SIS, MQOL-Physical symptoms, MQOL-Psychological symptoms, MQOL-Social support, and MQOL-Total score were statistically significantly lower in patients with ALS with fatigue compared with patients without fatigue (*p* = 0.007, 0.020, < 0.001, 0.004, and 0.004, respectively). There were no statistical differences between the two groups regarding MQOL-Physical wellbeing and MQOL-Existential wellbeing (*p* > 0.05).

Then, age, disease duration, the ALSFRS, ALSSS, ALSSSI + II, ALSSSIII + IV, HARS, HDRS, PSQI, ESS, and FSS scores were considered in the multiple linear regression model to find the impact of clinical variables on QoL. The variables included in the final regression models were determined by the screening step by step in the section of statistical analysis. The findings are shown in Table 6.

**Table 6:** The impact of clinical variables on quality of life in the multiple linear regression analysis.

	**Variables**	**B**	**Beta**	**95% CI**	***p*-Value**
MQOL-SIS	ALSSS (LE + UE)	0.202	0.312	(0.104, 0.301)	<0.001
	HDRS	−0.065	−0.206	(−0.112, −0.017)	0.008
MQOL-PhS	HARS	−0.193	−0.457	(−0.249, −0.138)	<0.001
	Age	−0.037	−0.146	(−0.071, −0.004)	0.030
MQOL-PhWB	HDRS	−0.125	−0.300	(−0.186, −0.063)	<0.001
	ALSSS (LE + UE)	0.235	0.274	(0.109, 0.362)	<0.001
MQOL-PsyS	HDRS	−0.085	−0.237	(−0.152, −0.017)	0.014
	HARS	−0.094	−0.252	(−0.162, −0.026)	0.007
	FSS	−0.220	−0.167	(−0.404, −0.035)	0.020
MQOL-EWB	HARS	−0.112	−0.348	(−0.157, −0.067)	<0.001
MQOL-SS	HARS	−0.061	−0.196	(−0.107, −0.015)	0.009
MQOL-Total	HARS	−0.271	−0.366	(−0.404, −0.138)	<0.001
	HDRS	−0.159	−0.226	(−0.287, −0.032)	0.014

*MQOL, McGill Quality of Life Questionnaire; MQOL-SIS, MQOL Single-Item Scale; MQOL-PhS, MQOL-Physical symptoms; MQOL-PhWB, MQOL-Physical wellbeing; MQOL-PsyS, MQOL-Psychological Symptoms; MQOL-EWB, MQOL- Existential wellbeing; MQOL-SS, MQOL-Social Support; ALSSS, Amyotrophic Lateral Sclerosis Severity Scale; LE, lower extremity; UE, upper extremity; HDRS, Hamilton Depression Rating Scale; HARS, Hamilton Anxiety Rating Scale; FSS, Fatigue Severity Scale. A multiple linear stepwise regression model was used*.

## Discussion

In this study, we found that fatigue was more frequent in patients with ALS than in HCs, although the frequency of fatigue in our patients was a litter lower than the previous reports (4–7). A previous study found that patients with ALS with fatigue were younger and had a longer duration of disease (5), which was absent in our observation. However, in contrast to previous negative results (5), significant differences between the two groups were observed regarding physical function, depression, anxiety, sleep and daytime sleepiness, and physical and psychological aspects of QoL.

After regression analysis excluding potentially confounding factors, the ALSSS (LE + UE), the ESS, and daytime dysfunction were associated with the presence of fatigue in patients with ALS, further proving their relationships with a higher risk of fatigue occurrence. Also, we found there was a relationship between ALSSS (LE + UE), daytime dysfunction, HDRS, and fatigue intensity. Regarding fatigue intensity-related factors, there have been controversial results among different studies. One study found that there was no significant correlation between the severity of fatigue and the other studied parameters (gender, site of onset, disease duration, functionality measured by the ALSFRS, depression, and sleepiness) (5). Another study also reported that the disease severity measured by ALSFRS, muscle strength, or disease duration did not correlate with the scores in the Multidimensional Fatigue Inventory (MFI) (4). However, on the contrary, the lower ALSFRS-R and depression were reported to be significant predictors of greater fatigue, evaluated by the same FSS as ours (6, 11), which was in consistent with the positive correlation between the HDRS and the intensity of fatigue found in our patients.

Though the lack of a correlation between the ALSFRS and fatigue, we found the ALSSS (LE + UE) and daytime dysfunction were related to fatigue intensity in our patients, which have never been reported. In patients with ALS, functionality was lost progressively with disease evolution and can have significance in the origin of fatigue. Moreover, weakness was an important complaint in the majority of patients with ALS (5) and muscular alterations due to disuse were one of the underlying mechanisms accounting for the etiology of fatigue in ALS (24). Considering the ALSSS (LE + UE) score reflected the function status of upper and lower limbs in patients with ALS. Therefore, in this study, we tended to attribute fatigue to dysfunction of limbs partly, resulting in disuse, which usually resulted from muscle weakness and atrophy of limbs.

As for daytime dysfunction, it referred to how often people have trouble staying awake and how much of a problem has it been for people to keep up enough enthusiasm to get things done (18). Theoretically, the emergence of fatigue in patients with ALS could result from poor quality sleep and EDS (5) and the FSS score has been reported to correlate with the ESS and the PSQI scores in patients with ALS (11). So, by analyzing the occurrence and intensity of fatigue, we clearly demonstrated the ALSSS (LE + UE) and daytime dysfunction were the most important influencing factors related to fatigue. Up to now, different studies have found relationships between various variables and fatigue intensity, and these discordances could arise from differences in the populations studied, in the methodology of the study, the size and selection of the samples, and different assessment scales used (5).

A previous study has indicated that patients with ALS described their QoL as involving physical, psychological, and other aspects (25). Considering both physical and psychological aspects of QoL in patients with ALS jointly facilitated a greater understanding of how clinical variables affect QoL. A multidimensional tool, the MQOL questionnaire, was adopted to assess QoL. The MQOL questionnaire had been widely used in patients with ALS (4, 26–29). Previously, it was indicated that age had a negative correlation with the subscores of the physical symptoms in the MQOL (4), in accord with our result. Also, the disease severity as measured by the ALSFRS and disease duration did not correlate with any domains in the eMQOL, consistent with the previous report (4). However, we found that the ALSSS (LE + UE) was positively related to the MQOL-SIS and the MQOL-physical symptoms. It was well-known that the MQOL-SIS was a single-item scale, evaluating the overall QOL and the MQOL-physical wellbeing domain indicated physical strength. The ALSSS (LE + UE) score reflected the spinal function status (upper and lower limbs) in patients with ALS. That was, the functional status of limbs may be the primary cause that can impact the overall QOL and physical strength in our patients.

Moreover, a former study reported that general fatigue, but not physical fatigue, contributed to the subscores of the physical symptoms in the MQOL, indicating psychological symptoms such as depression may affect the physical symptoms aspect of QOL (4). In this study, MFI was adopted for measuring five dimensions of fatigue independently in 25 patients with ALS: general fatigue, physical fatigue, mental fatigue, reduced motivation, and reduced activity. The FSS, another different instrument for fatigue evaluation, which focused solely on the physical symptoms of fatigue, was used in our study. A negative correlation between the FSS score and psychological symptoms of the MQOL was found. Considering the possible relationship of psychological symptoms with the physical symptoms aspect of QoL, our findings seemed to suggest physical fatigue may also affect the psychological symptoms aspect of QOL.

Depression scores were negatively correlated with QoL in patients with ALS, namely, the MQOL-SIS, physical wellbeing, psychological symptoms domains, and the MQOL-Total. Previous research has reported depression scores can significantly decrease QoL, in both physical and psychological domains (4, 25). It is worth noting that eight-four patients (48%) and 11 patients (6.3%) with ALS were in possible depression and depression status in our study, respectively, suggesting that subthreshold scores for depressive symptoms may also be actively treated due to their negative relationship with the QoL of patients (30).

Concerning anxiety, we found an inverse relationship of anxiety with QoL, consisting of physical symptoms, psychological symptoms, existential wellbeing, social support domains, and the MQOL-Total. Anxiety can undermine QoL in patients with ALS (31). A former study, consisting of 60 patients with ALS, found that depression and anxiety, assessed by the HADS, were associated with the psychosocial score (9). Another study with a much larger sample of patients reported anxiety was highly correlated with psychological QoL and mildly correlated with physical QoL (25). Besides, among patients with ALS, satisfaction with social relationships was found to be inversely correlated with anxiety (32).

As a whole, we found disease severity, depression, anxiety, age, and fatigue, especially depression and anxiety, were correlated with all the five domains of the MQOL, further confirming that QOL in patients with ALS is not only correlated with functionality but also with existential, psychological, and social support aspects (29).

There were some advantages in this study that should be indicated. First, this study was conducted to comprehensively characterize fatigue and to examine fatigue-associated factors and their impact on QoL in a large sample of Chinese patients with ALS. The assessment tools used in our study were brief and easily administered, allowing a simple and straightforward implementation in everyday clinical practice. Second, in addition to previously reported fatigue-related factors, such as functionality severity, depression, poor quality of sleep, excessive daytime somnolence, dyspnea (5, 6, 8, 11, 12), we added anxiety as another potential factor for analysis. Furthermore, a previous study found nocturnal complaints were significantly associated with fatigue in patients with ALS (11). Here, we identified daytime dysfunction, a specific aspect of the PSQI, can affect fatigue in this study. Most important of all, we found that the ALSSS (LE + UE) was related to fatigue, which has never been reported. Considering the ALSSS (LE+UE) score was representative of the functional status of limbs in patients with ALS. Therefore, dysfunction of limbs, which usually resulted from muscle weakness and atrophy of limbs, may account for fatigue in this study. Lastly, as was known, there was a general consensus that QoL in patients with ALS does not depend only on the level of physical impairment but was, rather, part of a complex reaction to several elements, including the level of social and financial support, psychological, and existential factors. Therefore, a multidimensional measurement of the MQOL was used in our study to examine QOL, finding the adverse impact of fatigue on the psychological symptoms of QOL in patients with ALS.

The main limitation of this study was as follows: first, it was an observational study design, lacking follow-up evaluation of fatigue in patients with ALS, so, we could not determine whether the effect on QoL varied over time or the effective treatments for fatigue, anxiety, and depression symptoms could improve QoL. Concerning this disease being uncurable now, fatigue and mood disturbance may offer other potential treatment targets for improving QoL. Whether effective management of those non-motor symptoms can help improve QoL in patients with ALS, deserved further study. Then, in the analysis, though we added dyspnoea as a potential influencing factor, which can contribute to fatigue in patients with ALS for some instances, however, objective measures of respiratory muscle function impairment, such as forced vital capacity, were not included in our analysis (11). Also, another limitation of the study was that medications (such as riluzole) may play a role in fatigue (8). Whereas, regarding the use of riluzole was variable and inconsistent in our patients due to the expensive price, the possible effects of drug use were not considered in our study. Lastly, although FSS was also a commonly used instrument to measure the severity of fatigue among patients with ALS, it focused solely on the physical symptoms of fatigue, so we could not measure physical and mental fatigue independently (8).

In conclusion, this study found that fatigue was commonly reported in Chinese patients with ALS. Also, functional status of limbs and daytime dysfunction were firstly identified as potential risk factors for fatigue. Notably, it highlighted the adverse effects of fatigue intensity on the psychological domains of QoL. As a consequence, clinicians should pay special attention to fatigue assessment for patients with ALS in clinical practice and treat it appropriately when necessary.

## Data Availability Statement

The original data in this study are available from the corresponding author on reasonable request.

## Ethics Statement

This study was approved by the Institutional Ethics Committee of West China Hospital, Sichuan University (approval 2020-942). The patients/participants provided their written informed consent to participate in this study.

## Author Contributions

RA, CL, XL, YW, XH, and SA performed material preparation, data collection, and analysis. RA wrote the first draft of the manuscript. CH and YX designed the research project and revised the manuscript. All authors contributed to the conception and design of the study, commented on previous versions of the manuscript, read, and approved the final version of the manuscript.

## Funding

This study was supported by the a platform of resource collection and standardized diagnosis and treatment for neurogenetic degeneration diseases (2019JDPT0015), the Post-Doctor Research Project, West China Hospital, Sichuan University (2020HXBH145), and Key research and development projects, Department of Science and Technology of Sichuan province (2021YFS0223).

## Conflict of Interest

The authors declare that the research was conducted in the absence of any commercial or financial relationships that could be construed as a potential conflict of interest.

## Publisher's Note

All claims expressed in this article are solely those of the authors and do not necessarily represent those of their affiliated organizations, or those of the publisher, the editors and the reviewers. Any product that may be evaluated in this article, or claim that may be made by its manufacturer, is not guaranteed or endorsed by the publisher.
